# Correlation between blood magnesium and calcium concentration in patients treated with an anti-EGFR antibody

**DOI:** 10.1186/s40780-016-0060-9

**Published:** 2016-09-22

**Authors:** Toshiyasu Tsujii, Takafumi Ogaki, Kaori Nakae, Kiyotaka Imai, Daisuke Kise, Shoji Tada, Hiroki Ueda, Masahiro Moriyama

**Affiliations:** 1 Section of Clinical Pharmaceutics, Department of Clinical Pharmacy, Graduate School of Pharmacy, Hyogo University of Health Sciences, 1-3-6, Minatojima, Chuo-ku, Kobe, Hyogo 665-8530 Japan; 2 Department of Pharmacy, Toyooka Public Hospital, 1094, Tobera, Toyooka, Hyogo 668-8501 Japan

**Keywords:** Hypomagnesemia, Calcium, EGFR-targeting antibodies, Cetuximab, Panitumumab

## Abstract

**Background:**

Hypomagnesemia is one of the characteristic side effects of the human anti-epidermal growth factor receptor (EGFR) monoclonal antibodies, cetuximab and panitumumab.

The major mechanism of anti-EGFR antibody-related hypomagnesemia is suppression of EGFR-mediated urinary Mg^2+^ reabsorption in both the renal tubule the intestinal tract.

Since Mg^2+^ is known to affect blood Ca^2+^ levels through regulation of parathyroid hormone (PTH) secretion, we investigated the correlation between Ca^2+^ and Mg^2+^ concentration in blood.

**Methods:**

Between April 2012 and October 2015, blood Mg^2+^ and Ca^2+^ concentrations (albumin corrected value) of 22 colon cancer patients undergoing treatment with either cetuximab or panitumumab at Toyooka Public Hospital were measured simultaneously.

**Results:**

Hypomagnesemia (of all Grades) was reported in 13 of 22 patients. Two patients had hypomagnesemia of severity > Grade 3.

Changes in blood Mg^2+^ and Ca^2+^ concentration showed a significant correlation (r^2^ = 0.7455), which could be expressed using the following equation, Ca^2+^ concentration = 1.4268 × (Mg^2+^ concentration) + 7.1126.

**Conclusion:**

Since the early stages of hypomagnesemia produce no characteristic clinical symptoms, it is easily overlooked until it becomes severe.

The investigation results suggest that if low blood Ca^2+^ concentration (mg/dL) is observed in patients administered anti-EGFR antibodies, early evaluation of blood Mg^2+^ concentration (mg/dL) and prompt supportive care are required to prevent aggravation of hypomagnesemia.

## Background

Cetuximab and panitumumab are anti-epidermal growth factor receptor (EGFR) monoclonal antibodies. Both antibodies are molecular-targeted drugs and their efficacy has been indicated not only in primary treatment, but also in secondary and tertiary treatment of advanced or recurrent colorectal cancer not harboring mutations in the KRAS gene [1].

Various side effects of molecular-targeted agents are known, which are unlikely to occur with a conventional cytotoxic agent. Some of the characteristic side effects of anti-EGFR antibodies include infusion reaction, skin symptoms such as acne-like rash, and electrolyte abnormalities. Of the electrolyte abnormalities, hypomagnesemia is a hidden side effect because it is asymptomatic and progresses slowly despite its high frequency [2].

The causes include inhibition of EGFR-mediated Mg^2+^ re-absorption from urine in renal tubules, and inhibition of EGFR-mediated Mg^2^+ absorption in the intestinal tract [3].

Furthermore, Mg^2+^ has been reported to affect blood Ca^2+^ level [4] via its in vivo control of parathyroid hormone (PTH) secretion.

Therefore, we examined the correlation between Ca^2+^ and Mg^2+^ concentration in patients’ blood.

## Methods

### Patients

The blood Mg^2+^ and Ca^2+^ concentrations (albumin corrected value) of 22 colon cancer patients who received an anti-EGFR antibody (either cetuximab or panitumumab) at our hospital from April 2012 to October 2015 were simultaneously measured. Fourteen of the 22 patients were treated with cetuximab. The remaining 8 patients received panitumumab (Table 1).

**Table 1:** Patient characteristics

*n* = 22		Mean ± S.D.	Median	Range
Age (year)		66.09 ± 7.78	65	51–80
Sex				
Male	17			
Female	5			
Body Height (cm)		162.84 ± 8.35	163.35	149.00–175.30
Body Weight (kg)		58.14 ± 9.11	56.55	40.00–74.00
PS				
0	14			
1	6			
2<	2			
Type of cancer				
Colon	13			
Rectal	7			
Cecum	2			
EGFR-targeting antibodies				
Cetuximab	14			
Panitumumab	8			
No metastatic sites				
One	7			
Two	8			
Three or more	7			
Involved sites	6			
Lung	13			
Liver	14			
Lymph nodes	3			
Bone	0			
Peritoneal	7			
Median treatment cycle			9.30	1.00–23.00
Median treatment duration, weeks			9.00	2.00–46.00
Combination of Magnesium oxide formulation				
Yes	11			
No	11			
Combination regimen				
FOLFIRI base	15			
FOLFOX base	2			
CPT-11	2			
Monotherapy	3			
Serum Magnesium (mg/dL)		2.13 ± 0.22	2.00	1.80–2.60
Serum Calcium (mg/dL)		9.96 ± 0.33	9.85	9.50–10.50
Laboratory data				
Neu (/mm3)		3716.8 ± 1459.1	3475.0	1380.0–6880.0
WBC (/mm3)		5936.3 ± 1685.9	5450.0	3200.0–10100.0
PLT (/mm3)		207,091 ± 5651	205,500	111,000–330,000
Hb (g/dl)		11.67 ± 1.73	11.55	7.80–14.4
sCre (mg/dl)		0.73 ± 0.31	0.64	0.51–1.88
AST (IU/l)		20.40 ± 4.44	21.00	12.00–28.00
ALT (IU/l)		15.40 ± 7.08	13.50	5.00–30.00

Patients treated with anti-EGFR antibodies = 43; serum Ca level was measured for all patients

### Procedures

Mg^2+^ and Ca^2+^ concentration in the blood was investigated retrospectively using information from electronic medical records. The severity of Mg^2+^ and Ca^2+^ concentration decline (hypomagnesemia/hypocalcemia) was graded according to Common Terminology Criteria for Adverse Events ver4.0 (CTCAE ver4.0 Japanese translation JCOG version).

Magnesium oxide formulations are commonly used as laxatives. Since they are expected to affect Mg^2+^ concentrations in blood, the incidence of hypomagnesemia was studied for magnesium oxide co-treated and magnesium oxide non-treated groups separately.

Skin toxicity is one of the characteristic side effects of human anti-EGFR monoclonal antibodies. Therefore, the relationship between hypomagnesemia and skin toxicity was investigated. Skin toxicities included pruritus, acneiform dermatitis, skin desquamation, exfoliative dermatitis, paronychia, nail disorder, skin fissures, skin laceration, pruritic rash, pustular rash, skin infection, and skin ulceration. Selected skin toxicities were graded according to Common Terminology Criteria for Adverse Events ver4.0 (CTCAE ver4.0 Japanese translation JCOG version).

Differences in patient background between the hypomagnesemia groups and non-hypomagnesemia groups were investigated.

### Statistical analysis

Statistical analyses were performed by using the chi-square test for categorical variables and Mann-Whitney’s *U* test for order variables. The relationship between Mg^2+^ and Ca^2+^ concentration in blood was examined using a single regression analysis. Statistical analysis was performed using SPSS® version 11. The significance level of the test was set at 5 %.

### Ethical issues

This study was approved by the research ethics committee of Toyooka Public Hospital (No. 102) and was performed according to the Declaration of Helsinki.

The waiver of informed consent from individual patients was approved by the ethics committee. Anonymized data with serial study ID numbers created by the study hospital were used throughout the study.

## Results

### Patients

Patient characteristics are shown in Table 1. Forty-three patients were treated with anti-EGFR antibodies. Of these, 21 patients were excluded from analysis owing to lack of data on Mg^2+^ concentration or asynchronous measurement of Ca^2+^ and Mg^2+^. Finally, 22 patients were included (17 men and 5 women); the median age was 65 years.

Mg^2+^ concentration before introducing an anti-EGFR antibody was 2.13 ± 0.22 mg/dL. Eleven patients received magnesium oxide co-treatment and the other 11 did not.

### Investigation of incidence frequency

The incidence frequency of hypomagnesemia after the start of anti-EGFR antibody administration was 59.1 % (13 cases). About 18.2 % of the patients (4 cases) had Grade 2 hypomagnesemia, and 9.1 % (2 cases) had Grade 3 hypomagnesemia.

The incidence frequency of hypocalcemia after the start of anti-EGFR antibody administration was 72.7 % (16 cases), with 4.5 % of patients (1 case) having Grade 2 hypocalcemia, and none having hypocalcemia higher than Grade3 (Table 2).

**Table 2:** Incidence of hypomagnesemia and hypocalcemia after treatment with cetuximab or panitumumab

	Hypomagnesemia *N* (%)	Hypocalcemia *N* (%)
Any Grade	13 (59.1)	16 (72.7)
Grade 2	4 (18.2)	1 (4.5)
Grade 3/4	2 (9.1)	0 (0.0)

### Correlation between Mg^2+^ and Ca^2+^ concentration in blood

**Fig. 1:**
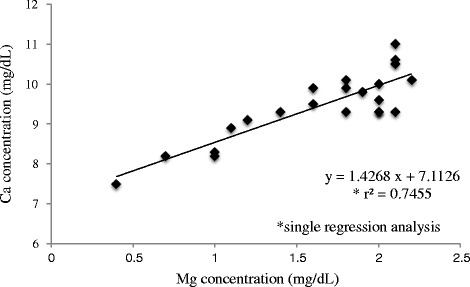
Correlation between individual serum Mg level and serum Ca level. The solid line indicates the linear regression line

Statistical analyses were performed using single regression analysis. The results show the trends and a significant correlation (r^2^ = 0.7455) between serum Mg^2+^ concentration and serum Ca^2+^ concentration that fits the following linear equation: Ca concentration = 1.4268 × (Mg concentration + 7.1126) (Fig. 1).

### Comparison of the incidence of hypomagnesemia in the presence or absence of oral magnesium oxide co-treatment

During treatment with anti-EGFR antibodies, 11 patients were co-treated with oral magnesium oxide preparation for constipation (group with co-treatment). The incidence of Grade 1 or higher hypomagnesemia in this group was 63.6 % (7 patients). The incidence in the magnesium oxide non-treated group (11 patients) was 54.5 % (6 cases).

**Fig. 2:**
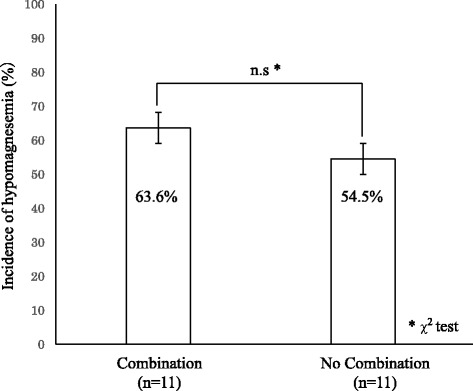
Oral magnesium oxide formulation administered to patients with hypomagnesemia during cetuximab and panitumumab treatment

No significant difference in incidence of hypomagnesemia was observed between the magnesium oxide co-treated group and the magnesium oxide non-treated group (Fig. 2; *p* = 0.665 by *χ*^2^ test).

### Correlation between severity of skin toxicity and hypomagnesemia

In anti-EGFR antibody-treated patients, the incidence of Grade 0 or 1 skin toxicity was 40.9 % (9 patients) and that of Grade 2 or higher skin toxicity was 59.0 % (13 patients). Magnesium values in each group were 1.80 ± 0.32, and 1.51 ± 0.60 mg/dL. No significant difference was observed (*p* = 0.53; Table 3).

**Table 3:** Correlation between the severity skin toxicity and hypomagnesemia

		Skin toxicity		
		Grade 0 ∙ 1	Grade 2<	*p
No. (%)		9 (40.90)	13 (59.00)	
Mg (mg/dl)	Mean ± S.D.	1.80 ± 0.32	1.51 ± 0.60	0.53

*Mann-Whitney’s *U* test

### Comparison of patient characteristics in hypomagnesemia

No significant difference in patient characteristics was observed between patients with and without hypomagnesemia (Table 4).

**Table 4:** Subset analyses of hypomagnesemia

		Hypomagnesemia		
		Grade 0	Grade 1<	*p*
No		9	13	
Sex^a)^	Men/Women	6/3	11/2	0.316
Body Hight (cm)^b)^	Mean ± S.D.	160.5 ± 9.6	164.4 ± 7.3	0.556
Body Weight (kg)^b)^	Mean ± S.D.	57.3 ± 7.7	58.6 ± 10.2	0.647
PS^a)^	0/1or 2	6/3	7/6	0.439
AST (IU/l)^b)^	Mean ± S.D.	20.1 ± 3.3	20.6 ± 5.1	0.794
ALT (IU/l)^b)^	Mean ± S.D.	14.4 ± 7.3	16.0 ± 7.1	0.695
sCre (mg/dl)^b)^	Mean ± S.D.	0.65 ± 0.10	0.79 ± 0.39	0.794
Neu (/mm^3^)^b)^	Mean ± S.D.	3253 ± 907	4037 ± 1704	0.431
WBC (/mm^3^)^b)^	Mean ± S.D.	5211 ± 1230	6438 ± 1815	0.144
PLT (/mm^3^)^b)^	Mean ± S.D.	193,778 ± 43,260	216,308 ± 64,165	0.556
Hb (g/dl)^b)^	Mean ± S.D.	11.83 ± 1.67	11.56 ± 1.83	0.948
Mg (mg/dl)^b)^	Mean ± S.D.	2.14 ± 0.25	2.12 ± 0.21	0.749
Ca (mg/dl)^b)^	Mean ± S.D.	10.00 ± 0.37	9.93 ± 0.32	0.845
Combination regimen	FOLFIRI base	6	9	
	FOLFOX base	0	2	
	CPT-11 alone	2	0	
	Monotherapy	1	2	

^a)^χ^2^test, ^b)^Mann-Whitney’s *U* test

## Discussion

In vivo, approximately 70 % of extracellular Mg^2+^ is re-absorbed by the ascending limb of the loop of Henle. The rest is re-absorbed in the proximal and distal tubules. Since EGFR is frequently expressed on the surface of the cells within the ascending limb of the loop of Henle, anti-EGFR antibodies such as cetuximab and panitumumab, inhibit EGFR in renal tubular epithelial cells [5]. Consequently, the expression level of the Mg^2+^ channel, TRPM6 (transient receptor potential member-6) decreases. Thus, Mg^2+^ transport decreases, resulting in Mg^2+^ loss in the kidney, which is believed to underlie the pathogenesis of hypomagnesemia [6, 7].

The intermediate aggregate results for a domestic performance survey of the anti-EGFR antibody cetuximab showed that electrolyte abnormalities occurred in 11.6 % patients (cases of Grade 3 or higher abnormalities, 1.6 %; and severe cases, 0.2 %). Of the total 1767 cases, 164 patients (9.3 %) had hypomagnesemia, 35 (2.0 %) had hypocalcemia, and 19 (1.1 %) had hypokalemia.

The results of cetuximab post-marketing all-patient surveillance as of July 2010 showed that among the 4345 total cases, 40 (0.9 %) exhibited decreased blood Mg^2+^, which is considerably smaller than that observed in Phase II clinical trials in Japan (51.3 %).

Since serum Mg^2+^ levels are not routinely measured at many facilities, the ability to detect hypomagnesemia may vary.

This varied capability can be considered a possible reason for the frequency gap between these two reaction reports. In vivo, Mg^2+^ participates in activation of enzymes, peripheral nerve regulation of muscle relaxation, and bone formation. Mg2+ also plays a particularly important role as a cofactor required for the catalytic activity of enzymes involved in intracellular metabolism. Although mild Mg^2+^ decrease is usually asymptomatic, as hypomagnesemia progresses, tetany, tremors, seizures, muscle weakening and other neuromuscular symptoms, psychiatric symptoms, cardiovascular symptoms such as severe arrhythmia, and other electrolyte abnormalities occur as complications [8]. Since hypomagnesemia is difficult to detect from subjective symptoms in its early stages, few complaints have been reported. Therefore, regular measurement of serum Mg^2+^ level is needed.

In this study, the effect of administration of magnesium oxide preparations in combination with an anti-EGFR antibody was also analyzed. In the group administered magnesium oxide preparations, although hypomagnesemia improvement occurred, no relation was observed between magnesium oxide administration and hypomagnesemia improvement. Since oral magnesium oxide preparations are only weakly absorbed in the intestinal tract and are excreted by absorbing moisture from the intestine, the lack of correlation could be attributed to its low oral bioavailability.

In addition, skin toxicity such as acne-like rash and paronychia with anti-EGFR antibody treatment, can lead to early discontinuation of treatment. But Anti-EGFR antibody drugs have been reported to have a marked therapeutic effect in patients who exhibit skin toxicities. Using the data in the present study, we investigated the correlation between skin toxicity and electrolyte disturbances. We also looked for correlations between patient characteristics and electrolyte disturbances, but were unable to discover a significant correlation. Since all patients are routinely and thoroughly screened for skin toxicity symptoms, we consider any effect of skin toxicities on electrolyte imbalances (or vice versa) to be slight.

Since an anti-EGFR antibody can now be used as the primary treatment, cases of chronic administration are increasing. Further, in cases where hypomagnesemia has been reported, prolonged treatment with cetuximab is currently required because of its marked therapeutic effect^8)^. It has been reported that in the elderly and in patients with high pre-treatment serum Mg^2+^ levels, the risk of developing hypomagnesemia is high, and the frequency of severe hypomagnesemia increases if anti-EGFR drug administration exceeds 6 months [9]. Nakamoto et al. reported that since recovery from severe hypomagnesemia is poor even if supplemental Mg2+ is administered, withdrawal of anti-EGFR antibodies was situationally required. To prevent severe hypomagnesemia, prompt initiation of supportive care should also be required [10].

Hypercalcemia has been reported in about 20–30 % of cancer patients. In patients with malignant tumors, bone destruction and tumor cell expansion associated with bone metastasis lead to the production of PTH-related peptides, which eventually result in the development of hypercalcemia. Since onset is often sudden, blood Ca^2+^ concentration is regularly measured.

Hypercalcemia is typically associated with bone metastasis. Although all patients were at stage IV, a significant correlation between magnesium and calcium concentrations in bone metastatic cancer patients was not observed. ALP is a bone metastasis marker. A significant difference in changes in ALP levels was not observed between the hypercalcemia group and the non- hypercalcemia group (data not shown).

Ca^2+^ is a major regulator of PTH secretion, and Mg^2+^ regulates PTH secretion as well. Lack of intracellular Mg^2+^ is known to suppress PTH secretion, which may result in reduction of serum PTH and Ca^2+^ levels. Therefore, in patients with hypomagnesemia due to administration of anti-EGFR antibodies, hypocalcemia could be concurrent [4].

Since a correlation between Ca^2+^ and Mg^2+^ concentration in patients treated with an anti-EGFR antibody was indicated in this investigation, it is suggested that prompt evaluation of blood Mg^2+^ concentration is required to avoid incidence of hypomagnesemia, especially when reduction in blood Ca^2+^ concentration is detected.

## Conclusions

In conclusion, the investigation results suggest that if low blood Ca^2+^ concentration (mg/dL) is observed in patients administered anti-EGFR antibodies, early evaluation of blood Mg^2+^ concentration (mg/dL) and prompt supportive care are required to prevent aggravation of hypomagnesemia. Changes in blood Mg^2+^ and Ca^2+^ concentration showed a significant correlation (r^2^ = 0.74555), which could be expressed by the following equation: Ca^2+^ concentration = 1.4268 × (Mg concentration) + 7.1126. Since there is no characteristic clinical symptom in early stages of hypomagnesemia, it is easily overlooked until it becomes severe. Since the correlation between Ca^2+^ and Mg^2+^ concentration in patients treated with an anti-EGFR antibody was indicated in this investigation, it is suggested that prompt evaluation of blood Mg^2+^ concentration is required to avoid an incidence of hypomagnesemia, especially when reduction in blood Ca^2+^ concentration is detected.

## Abbreviations

ALP: Alkaline phosphatase
CTCAE ver4.0: Common terminology criteria for adverse events ver4.0
EGFR: Human anti-epidermal growth factor receptor
PTH: Parathyroid hormone
TRPM6: Transient receptor potential member-6
